# Umbilical Nodule Metastasis from Unknown Primary: Diagnostic and Therapeutic Dilemma

**DOI:** 10.1055/s-0042-1749423

**Published:** 2022-06-30

**Authors:** Sweety Gupta, Gaurav Sharma, Nidhi Sharma, Shreyosi Mandal, Amit Gupta, Manoj Gupta

**Affiliations:** 1 Department of Radiation Oncology, All India Institute of Medical Sciences, Rishikesh, Uttarakhand, India; 2 Department of Surgery, All India Institute of Medical Sciences, Rishikesh, Uttarakhand, India

**Keywords:** umbilical, metastases, palliative chemotherapy, unknown primary

## Abstract

Umbilical nodule metastasis is not a common presentation of carcinoma of unknown primary. It may be the presenting symptom of a primary malignancy or as metastatic site of previously diagnosed cancer and is considered to be a poor prognostic finding. We here report a case of young male who presented with umbilical mass, but the primary could not be localized even after thorough investigations and work up. Also, there existed therapeutic dilemma because immunohistochemistry did not favor one site, and so he was considered for combination chemotherapy in view of unresectable disease.

Metastatic carcinoma of unknown primary site (CUPS) constitutes 3 to 5% of all cancers. 1 These are labeled as unknown primary when, even after thorough clinical examination and elaborate diagnostic workup, such as imaging, histopathology including immunohistochemistry, the primary site cannot be ascertained. Most common sites of metastasis are liver, lung, bone, brain, lymph nodes (mainly cervical, inguinal, and axillary nodes), and peritoneum and pleura. 2 Umbilical metastases (UM) are uncommon and represent only 10% of all secondary tumors which have spread to the cutaneous region. 3 UM are also termed as the Sister Mary Joseph's nodule (SMJN) and are considered to be the sign of poor prognosis. Sir Hamilton Bailey in 1949 coined this term in honor of Sister Mary Joseph who was the first one to note the association between umbilical nodules and intra-abdominal malignancies. 4 Umbilical nodule metastases can occur from gastrointestinal (35–65%) and genitourinary (12–35%) tracts and hematological, lung, and breast (3–6%) cancers. Primary site of malignancy varies in males and females. 5 Umbilical lesion might be the only site of involvement in 5% of cases. 6 7 Median survival of patients with CUPS varies from 6 to 9 months. 8 We, here, report a case of young male in whom UM was the presentation and primary could not be identified even after comprehensive diagnostic work up. It has been 17 months since diagnosis and patient has received multiple lines of chemotherapy, with preserved performance status and less than partial response to chemotherapy.

## Case Report

**Figure 1:**
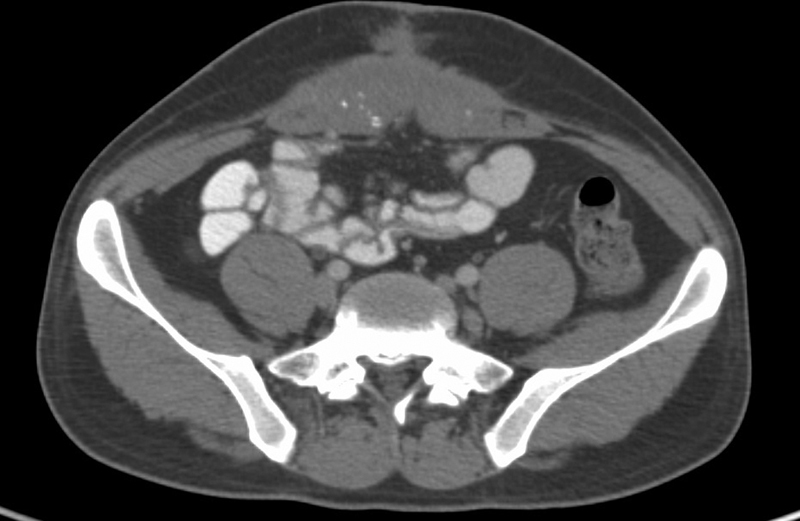
Contrast enhanced CT scan Abdomen showing umbilical lesion.

**Figure 2:**
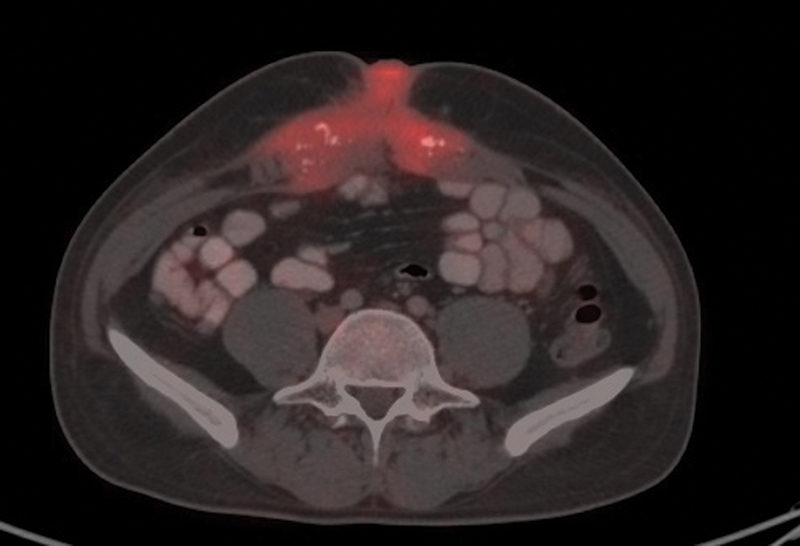
PET CT scan showing nodular lesion at umbilicus and omental nodules in sub hepatic region.

**Figure 3(a–d):**
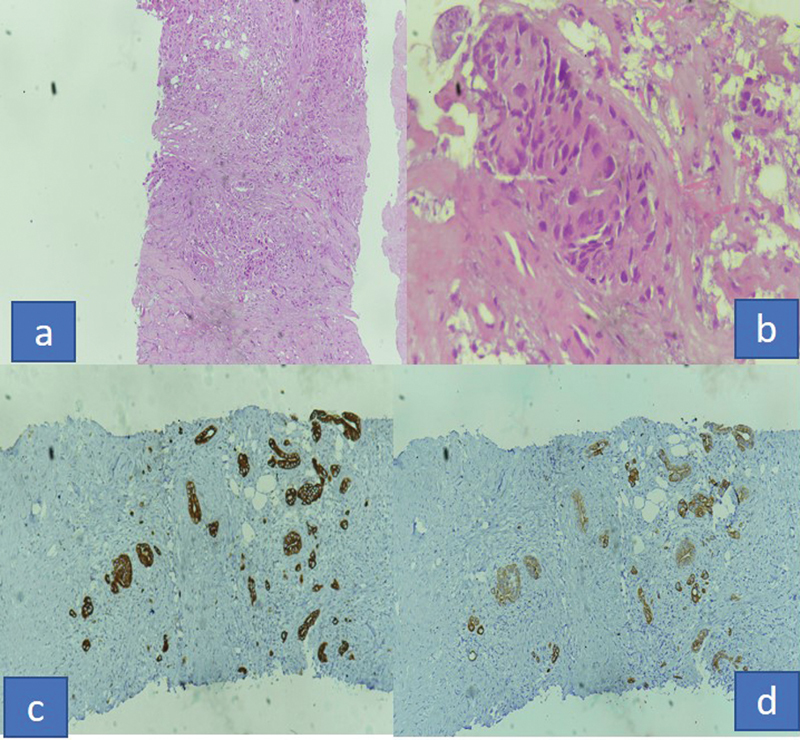
Microscopic photograph of biopsy from umbilical lesion (a) 10x (b) 40x hematoxylin section (c) IHC CK7 positive (d) CK 20 positive sections.

A 29-year-old male presented to oncology out patient department with complaints of pain in abdomen and passage of black colored stools for last 1 month. Pain was intermittent in nature, not associated with vomiting or food intake. On clinical examination, 8 cm × 7 cm hard, partially fixed mass was palpable in umbilical region. Routine blood parameters were normal. Serum Carcinoembryonic antigen (CEA) was 293 ng/mL. Contrast-enhanced computed tomography (CT) scan of abdomen and pelvis revealed 9.1 cm × 3 cm × 9 cm mass involving medial aspect of both the rectus muscles in midline at level of umbilicus, rectus sheath was infiltrated by lesion, with puckering of umbilicus and reaching up to the skin surface ( Fig. 1 ). His lower and upper gastrointestinal endoscopy were normal. Positron emission tomography (PET)-CT scan reported soft tissue density lesion in right anterior abdominal wall nodular lesion at umbilicus and omental nodules in subhepatic region ( Fig. 2 ) Core biopsy from the mass was suggestive of metastatic carcinoma. Immunohistochemistry was CK7, CK20, CK19, and 34BE12 positive and B-catenin diffuse cytoplasmic positivity in tumor cells with possibility of (1) primary from pancreaticobiliary or (2) urachal adenocarcinoma ( Fig. 3A–D ). In view of clinicoradiological findings, raised tumor marker and presence of omental metastases patient was planned for capecitabine and oxaliplatin (CAPOX) based palliative chemotherapy. He received four cycles of CAPOX but in view of radiological disease progression, he was started on taxane- and platinum-based chemotherapy. After six cycles of radiologically, it was a stable disease, so it was changed to taxane- and gemcitabine-based chemotherapy. After three cycles, there was disease progression, so chemotherapy was changed to FOLFIRI regimen. In view of poor tolerance to 5 fluorouracil, it was replaced by capecitabine and till date, he has received two cycles. Seventeen months after the diagnosis, patient is alive with disease and good performance status without having any symptoms due to disease.

## Discussion

CUPs are metastatic cancers with very poor prognosis in which the primary site could not be ascertained after a thorough evaluation and diagnostic work up. Common histopathologies include adenocarcinoma (60%), squamous (5%), neuroendocrine (5%), and poorly differentiated carcinoma (30%). 9 Immunohistochemistry may be required in conjunction with histopathology to identify primary site, for example, lymphoma and germ-cell tumors. Most common sites of metastases include liver, lung, bone, brain, lymph nodes, and pleural and peritoneal cavities with lung, bone, and abdomen sites occurring in elderly age groups. 10 Umbilical nodule metastasis is rare, and it, as a first sign of malignancy, is even more rare. It can be a primary tumor or metastasis from a primary elsewhere. It is seen in 15 to 29% of cases in absence of primary. 11 It can develop 1 to 12 months prior to diagnosis of primary site. 12 It indicates advanced stages of malignancy and is associated with poor prognosis. However, it has been seen that prognosis is better if umbilical nodule metastasis is diagnosed before the primary. 13 Usual presentation is a painful hard umbilical lump, surface may be necrotic or ulcerated. Size of the nodule varies from 0.5 to 2 cm, though some nodules may reach up to 10 cm in size. 14 Our patient also had size of nodule around 9 cm. Mechanism of spread have various hypothesis. Seeding process can occur through contiguous spread of peritoneal infiltration (the most common route) or through arterial, venous, or lymphatic channels. Spread via embryonic structures (such as urachus, round ligament of liver, vitello intestinal duct remnant, or obliterated vitelline artery) is also proposed possible method of spread. Other routes have been mentioned as specific to cancer, for example, urinary bladder malignancies through urachus. It has also been seen that most gastrointestinal malignancies with umbilical nodule also metastasize to liver and in those cases venous and lymphatic channels between liver and umbilicus are the most probable mechanism of seeding. 15 Most common histopathology in UM is adenocarcinoma and rarely a squamous carcinoma, melanoma, or sarcoma. Diagnostic tools used to characterize an umbilical mass are ultrasonography and abdominal CT scan. 16 All umbilical nodules should be biopsied to determine the primary histopathology. Presence of an umbilical metastasis portends a poor prognosis and is usually a sign of advanced malignancy. Survival of these patients without treatment ranges from 2 to 11 months as per available literature. 17 Some studies suggest that if UM is detected before primary malignancy management, the survival is better 9.7 months as compared with those in which lesion appears after the primary treatment has been done 7.6 months. 18 19 In our case, patient has survived for more than 17 months and is still alive with disease. Prognosis also depends on the primary tumor site and better survival rates have been reported with ovarian malignancy. 20 Also, patients who managed with combination of surgery and chemotherapy are fare better than patients who are candidates for palliative chemotherapy only. But treatment approach depends on the stage at presentation. In this case, diagnostic and therapeutic dilemma remained even after thorough radiological and pathological evaluation because primary site could not be identified so appropriate chemotherapy regime selection was complicated. Newer modalities for diagnosis include gene expression profiling by mRNA based assay that may assist in diagnosis in some subset of patients and guide toward use of targeted therapy. 21

## Conclusion

CUPs pose a diagnostic and management if primary site is not discernible even after complete diagnostic work up. Immunohistochemistry also may occasionally not suggest any putative primary site. In such cases, combination (surgery and chemotherapy) is the only option. Also, gene profiling assay may be considered to suggest primary site and consider for targeted therapy.
